# Trauma induces overexpression of Cx43 in human fetal membrane defects†

**DOI:** 10.1002/pd.5104

**Published:** 2017-08-01

**Authors:** David W. Barrett, Aumie Kethees, Christopher Thrasivoulou, Alvaro Mata, Alex Virasami, Neil J. Sebire, Alex C. Engels, Jan A. Deprest, David L. Becker, Anna L. David, Tina T. Chowdhury

**Affiliations:** ^1^ Institute of Bioengineering, School of Engineering and Materials Science Queen Mary University of London London UK; ^2^ Department of Cell and Developmental Biology University College London London UK; ^3^ Histopathology Department, Camelia Botnar Laboratories Great Ormond Street Hospital for Children NHS Trust London UK; ^4^ Department of Obstetrics and Gynaecology University Hospitals Leuven Leuven Belgium; ^5^ Lee Kong Chian School of Medicine Nanyang Technological University Singapore; ^6^ Institute for Women's Health University College London London UK

## Abstract

**Objective:**

We developed an *in vitro* model to examine whether trauma induces connexin 43 (Cx43) expression and collagen organisation in the amniotic membrane (AM) of fetal membrane (FM) defects.

**Method:**

Term human FM was traumatised *in vitro*. Cell morphology and Cx43 were examined in the wound edge AM by immunofluorescence (IMF) confocal microscopy and compared to control AM. Collagen microstructure was examined by second harmonic generation (SHG) imaging. Cell viability was assessed with calcein and ethidium staining.

**Results:**

After trauma, the AM showed a dense region of cells, which had migrated towards the wound edge. In wound edge AM, Cx43 puncta was preferentially distributed in mesenchymal cells compared to epithelial cells with significant expression in the fibroblast layer than epithelial layer (p < 0.001). In the fibroblast layer, the collagen fibres were highly polarised and aligned in parallel to the axis of the wound edge AM. There was an absence of cell migration across the defect with no healing after 168 h. Cell viability of the FM after trauma was maintained during culture.

**Conclusion:**

Cx43 overexpression in wounded AM drives structural changes in collagen that slows down efficacy of cell migration across the FM defect. © 2017 The Authors. *Prenatal Diagnosis* published by John Wiley & Sons, Ltd.

## Abstract 2

What's already known about this topic?

- After fetal surgery, the human fetal membrane shows limited healing and overexpression of Cx43 at the wound edge.
- Cx43 knockdown leads to accelerated wound healing by influencing cell migration and tissue dynamics.

What does this study add?

- We developed an artificial fetal membrane model to examine Cx43 expression after trauma and changes in collagen dynamics.
- We observed Cx43 overexpression and polarised collagen at the wound edge. These changes were found to be similar to human fetoscopic wounds after fetal surgery.

## Introduction

Iatrogenic preterm premature rupture of the fetal membranes (iPPROM) is a major complication after invasive fetal diagnostic or therapeutic fetal interventions and is a significant threat to fetal survival and well‐being if the membranes rupture preterm, before 37 weeks of gestation. iPPROM occurs in 6 to 45% of cases after fetoscopic procedures with complications related to the number of fetoscopic ports required, defect size, amniotic fluid (AF) leakage and membrane separation.^1^, ^2^, ^3^, ^4^ The high prevalence of iPPROM after fetal interventions reduces the effectiveness of interventions to treat fetal abnormalities.

Several research groups have explored potential therapies to prevent iPPROM or to repair the fetal membrane (FM) defect after established rupture. In pre‐clinical models, development of methods to seal the FM defect using collagen plugs or maternal platelets mixed with fibrinogen to promote adhesiveness of the amniopatch therapy was reported, but neither approach prolonged pregnancy with favourable perinatal outcome.^5^, ^6^, ^7^, ^8^ Other authors used a variety of tissue sealants to plug defects within explant or *in vivo* models albeit with varying success.^9^, ^10^, ^11^, ^12^, ^13^, ^14^, ^15^, ^16^ In contrast, studies of FM healing in animal models revealed limited repair of tissues in large mammals such as rhesus monkey or sheep.^17^, ^18^ This is in contrast to rats, where adhesion of the AM and chorionic membrane (CM) was observed 5 days after FM wounding, with fibroblast proliferation and clot formation, and in rabbits, restoration of tissue integrity was induced by matrix‐metalloproteinase (MMP) ‐2 and ‐9 activity.^19^, ^20^ Whilst the *in vitro* models have adopted a variety of tissue engineering approaches to induce healing after trauma and assess treatment efficacy using monolayer, explant or decellularised FM models, the evidence of healing the FM defect is variable with conflicting data on repair.^21^, ^22^

The FM is made up of the amniotic and chorionic layers consisting of multiple cell types embedded within a 3D extracellular matrix network.^23^ The differences in repair potential by cells present in the epithelial and fibroblast layers of the AM are primarily due to the absence of a vascular supply. This nature makes it difficult for the epithelial and mesenchymal cells to follow normal repair mechanisms and migrate to the site of injury to heal the defect. The gap junction protein connexin 43 (Cx43) has been shown to play a pivotal role early during the acute wound healing process in a variety of epithelial cell surfaces. In chronic skin wounds, Cx43 is overexpressed, and healing is perturbed due to delayed cell migration.^24^, ^25^, ^26^ We observed enhanced Cx43 in the AM surrounding fetoscopic wound defects, many weeks after fetoscopic laser surgery for twin‐to‐twin transfusion syndrome (TTTS). The presence of this protein in the AM induces morphological and structural changes in the collagenous matrix that has the potential to interfere with normal healing mechanisms.^27^ We hypothesise that Cx43 could affect cell migration, proliferation and collagen organisation in wounded AM and compromise membrane integrity. In the present study, we developed an artificial trauma model that mimics the creation of a fetoscopic surgical defect site in the human FM. We examined whether trauma of the FM induces changes in Cx43 expression and collagen architecture in the wound edge AM.

## Methods

### Patient recruitment, fetal membrane and amniotic fluid collection

Ethical approval was granted by the Joint UCL/UCLH Committees on the Ethics of Human Research (Ref: 08/H0714/87). Term human placentas (*n* = 12) were collected with informed consent from women undergoing elective caesarean section at University College Hospital London. Women with placenta praevia, multiple pregnancy, antepartum haemorrhage, PPROM and fetal growth restriction were excluded from the study. At Caesarean section, before delivery of the placenta, a clip was placed on the lower edge of the FM within the uterine incision to provide a landmark. The placenta was separated from the uterus by gentle cord traction for 3 min and rinsed with Earle's Balanced Salt Solution (EBSS) to remove excess maternal blood (Sigma‐Aldrich, Fancy Road, Poole, UK). FM was dissected into 30 × 30 mm specimens, washed with Earle's Balanced Salt Solution (EBSS) for 2 min and incubated in DMEM +20% FCS (Sigma‐Aldrich, UK). AF was collected from women undergoing fetoscopic laser ablation of placental vascular anastomoses for twin‐to‐twin transfusion syndrome in mid‐trimester pregnancies.^28^ Immediately after ultrasound‐guided placement of the fetoscope into the amniotic cavity, AF was withdrawn using sterile syringes (50–2000 mL).

### Artificial fetoscopic fetal membrane trauma model

The FM explant was assembled with the crown insert by clamping the tissue in between the crown body and ring. The FM was punctured using a 21 Gauge needle and held in place for 2 min to create a 0.8 mm diameter defect in the immobilised FM. The CellCrown™ inserts (Scaffdex Oy, Tampere, Finland) loaded with the FM specimen were subsequently transferred into the well of a tissue culture plate. Human AF (0.5 mL) was injected into the well to completely fill the area below the FM explant and cultured for 24 and 168 h with AF replaced every 48 h. Control membranes were taken from the same patient and loaded identically with no wound. At the end of the experiment, the control and wounded FM explants were fixed in 4% PFA and stored in PBS at 4 °C prior to analysis.

### Immunostaining

Immunostaining was performed with whole‐mount control and wound edge FM specimens that had been fixed in 4% PFA for 2 h and incubated with primary antibodies for Cx43 (diluted 1:4000, Sigma, 6219) at room temperature overnight, as described.^24^ The tissues were washed with PBS and incubated with goat Alexa 488 anti‐rabbit secondary antibody for 2 h at room temperature (1:400, Life Technologies). Secondary antibody incubation in the absence of primary antibody was used as a negative control. Tissues were counterstained for 20 min with 1 μg/mL of the nuclear dye DAPI (1:1000).

### Second harmonic generation and confocal imaging

Control and wound edge specimens were imaged in the AM tissue region by two photon imaging on a Leica TCS SP8 acousto‐optic beamsplitter (AOBS) multiphoton confocal laser scanning microscope (Leica, Milton Keynes, UK) with a Coherent Chameleon Ultra, Ti Sapphire mode‐locked IR laser (Coherent UK Ltd, Cambridge UK), as previously described.^27^ Briefly, samples were imaged with a 25×, 0.95 NA water‐immersion objective. The Cx43 signal (920‐nm excitation) was collected with the non‐descanned external HyD detector through an FITC emission filter (500 to 550‐nm barrier filter). The DAPI signal was collected sequentially with 405‐nm excitation to avoid bleed through of nuclear signal into the Cx43 signal and emission signal via the confocal pin‐hole to the de‐scanned HyD detector between 400 and 490 nm. Collagen SHG signal was collected via the transmission detector and 430 to 450‐nm barrier filter with a pump wavelength of 880‐nm at 80‐fs pulse width. Approximately 150‐μm volumes were acquired through the full thickness of the AM at 1.5 μm *z*‐section intervals. Parameters for laser power, detector gain and offset were kept constant for each sample so that direct comparisons of the 8‐bit digital images could be made per patient to permit quantification.

### Confocal image quantification and quantitative analysis

Cx43 immunostaining levels were quantitatively evaluated per tissue area and per cell nuclei using a well‐established pixel‐counting method in control and wounded AM.^27^ Maximum projections were performed in the AM to examine the cell populations in the fibroblast and epithelial layer. The images were converted to binary images using identical threshold values and objects exceeding 2 pixels were counted to identify Cx43 positive pixels per tissue area (μm^2^ per 0.25 mm^2^ tissue sections) or per cell nuclei. To characterise the direction of collagen alignment, an orientation distribution analysis using the directionality ImageJ plugin was performed. SHG images were converted to binary and 2D orientation analysis calculated using the local gradient orientation method.

### Histology

FM explants were fixed in 4% PFA and embedded in paraffin. Sections were haematoxylin and eosin (H&E) stained. Wound edge sections were identified from India ink stains.

### Analysis of cell viability

FM explants were incubated with 5 μM of calcein AM and 5 μM ethidium homodimer (Invitrogen, Paisley, UK) for 45 min at 37 °C and visualised with a 20× objective using an epifluorescence microscope. Cells were labelled as green (live) and red (dead) by calcein‐AM and ethidium homodimer, respectively. Percentage cell viability was calculated from at least ten fields of view.

### Statistical analysis

*Post hoc* Bonferroni‐corrected *t*‐test were used to examine data for Cx43 protein expression in control and wound edge FM. The number of replicates per patient for the control and wound edge FM are indicated in the figure legend. In all cases, a level of 5% was considered statistically significant (*p* < 0.05).

## Results

### Features of the amniotic membrane defect after trauma

**Figure 1:**
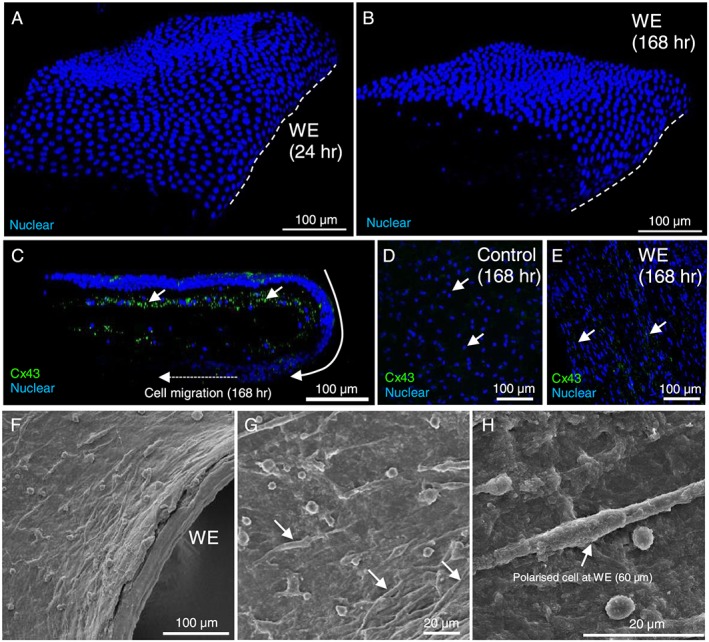
Cx43 expression and cell morphology in wounded amniotic membrane after trauma. Immunofluoresence confocal microscopy was used to examine Cx43 protein expression and cell morphology in the amniotic membrane (AM), after creation of a defect in the fetal membrane (FM) explant using the artificial FM trauma model. After 24 and 168 h of culture, representative images in (A) and (B) show epithelial curling at the wound edge (WE). After 168 h of culture, the cells have crawled forward to form a layer above and below the wounded specimen (C) and show typical punctate staining of Cx43 plaques (indicated by white arrows). Control specimens show rounded cell nuclei and negligible Cx43 in the fibroblast layer after 168 h of culture (D) compared to higher expression of Cx43 at the WE (E). SEM analysis of the wounded AM (F–H) confirmed polarised mesenchymal cell morphology tangential to the WE (G). The dotted white lines show the border along the length of the WE in the AM. Scale bar = 100 μm, unless indicated otherwise

Figure 1 shows cell morphology and Cx43 protein expression in the wound edge AM with full thickness defects created with a 21 Gauge needle and tissue explants cultured up to 196 h using the artificial FM trauma model. After 24 and 196 h of culture, we observed a distinctive blanket of cells with increased cell density in the wounded AM (Figure 1A, B, respectively). After 196 h of culture, the cells had migrated towards the centre of the wound edges and formed a thickened layer of flattened cells at the edge and outer tissue layer, in an attempt to seal off the wound (Figure 1C). Representative images of Cx43 in the wound edge AM show that the protein was distributed in a scattered and punctate fashion, with greater localisation in cells in the fibroblast layer compared to the epithelial layer (Figure 1C, E). In contrast, control AM show rounded cell nuclei and negligible Cx43 puncti in the fibroblast layer after 168 h of culture (Figure 1D). A common feature in cell morphology within wounded AM was the presence of mesenchymal cells that were polarised at 90° to the leading wound edge (Figure 1E–H). In contrast, cells away from the wound edge appeared round in morphology (Figure 1D).

### Cx43 protein expression was increased in the fibroblast layer of the amniotic membrane defect after trauma

**Figure 2:**
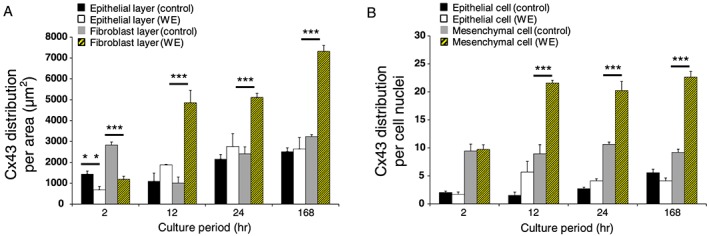
Cx43 protein distribution in the epithelial and fibroblast layer of the wounded amniotic membrane after trauma. The distribution of Cx43 was analysed per unit tissue area for comparisons between epithelial and fibroblast layer (A) and per cell nuclei (B) and compared to control and wound edge (WE) specimens. In all cases, error bars represent the mean and SEM values for *n* = 4 to 6 replicates, where membranes were taken from at least three patients. Significant differences were found as indicated by ***p < 0.001. All other comparisons were not statistically significant (not indicated)

We quantitatively compared the changes in Cx43 protein expression in the wounded AM with time in culture and compared to control membranes using a well‐established pixel counting method. After 2 h of culture, the levels of Cx43 protein distribution in the wounded epithelial and fibroblast layer of the AM were low and ranged from 686.5 to 1189.1 μm^2^, respectively (Figure 2). After 168 h of culture, the levels of Cx43 protein expression significantly increased in wounded AM with greater values in the fibroblast layer (7315.8 μm^2^) when compared to the wounded epithelial layer (2642.6 μm^2^) or control epithelial (2521.4 μm^2^) and fibroblast (3234.5 μm^2^) layers (all *p* < 0.001; Figure 2A).

### Mesenchymal cells preferentially express Cx43 in the amniotic membrane defect after trauma

**Figure 3:**
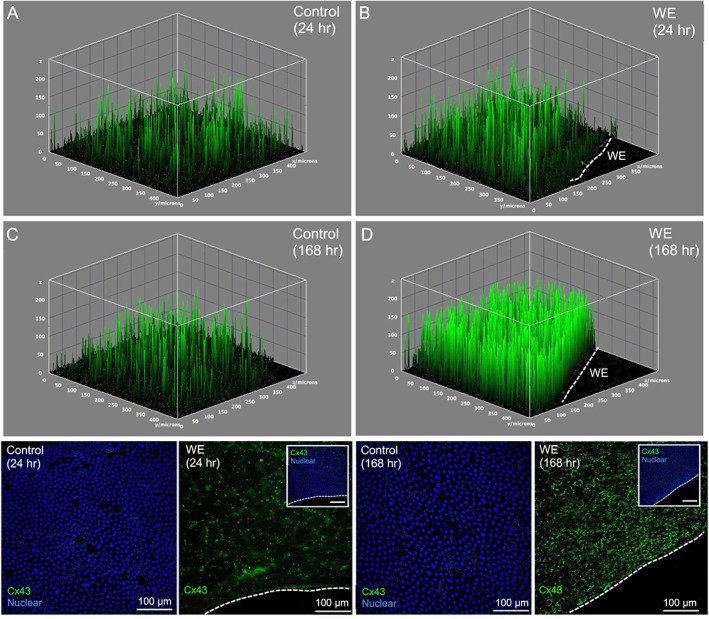
Cx43 distribution and plaque formation in the wounded amniotic membranes after trauma. The distribution of Cx43 plaque density was quantified in control and wound edge (WE) amniotic membranes (AM) cultured for 24 and 168 h. Control specimens show max projections and minimal expression of Cx43 by cells present in the epithelial and fibroblast layer (A, C) compared to wounded AM (B, D). Each spike represents a Cx43 plaque, and the dotted white lines show the border along the length of the WE in the AM. Merged images in bottom panel show characteristic spotted staining of Cx43 protein expression in the fibroblast layer of the WE. Blue signal is DAPI staining of nuclei and Cx43 in green detected by confocal imaging. Scale bars: 100 μm, inset shows corresponding merged image with nuclear staining

In wounded AM, quantitative analysis of Cx43 distribution per cell was significantly increased in mesenchymal cells (20.2 to 22.6 pixels/cell nuclei) when compared to amniotic epithelial cells (3.8 to 4.1 pixels/cell nuclei) after 12, 24 and 168 h of culture (all *p* < 0.001, Figure 2B). In contrast, values for Cx43 protein expression were relatively low in patient matched control epithelial (5.5 pixels/cell nuclei) and mesenchymal cells (9.2 pixels/cell nuclei) AM cultured for the same time period. Analysis of max projections by IMF confocal microscopy showed negligible Cx43 expression in control AM that were cultured for 24 and 168 h (Figure 3A, C), compared to a greater number of plaques distributed by cells present in the wounded AM (Figure 3B, D). Bottom panel in Figure 3 shows typical punctate plaques of Cx43 localised at the sites of cell–cell contacts by IMF confocal microscopy that was not observed in patient matched control AM.

### Collagen organisation in the amniotic membrane defect after trauma

**Figure 4:**
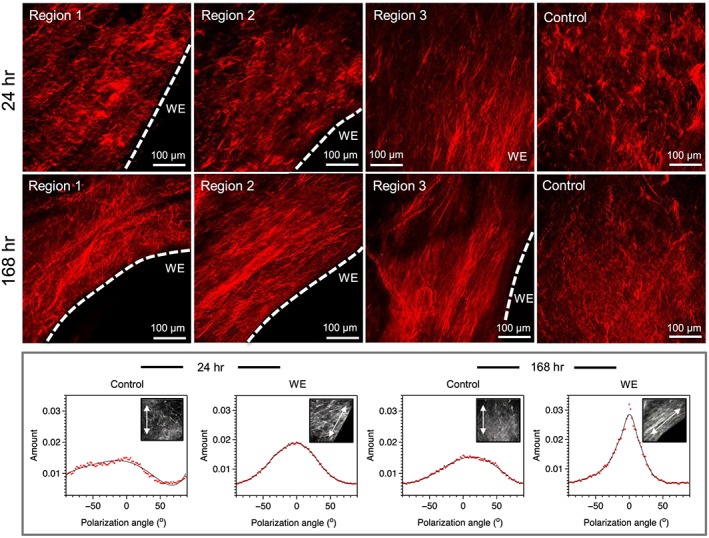
Collagen organisation in the wounded amniotic membranes after trauma. Second‐harmonic generation imaging of collagen (red) shows dense collagen fibre alignment tangential to the wound edge (WE) of the amniotic membrane (AM) in three separate regions, compared to disorganised and shorter collagen fibres in control AM taken away from the WE. Collagen fibre alignment at the leading WE appear to increase from 24 to 168 h, as shown by fibre alignment analysis (bottom panel), with control fibre alignment remaining unchanged. The collagen fibre alignment graphs are representative images and indicate the amount of collagen aligned at a specific polarisation angle. The dotted white lines show the border along the length of the WE in the AM. Scale bar = 100 μm

Control and wound edge AM were examined by SHG imaging to compare collagen structure in the matrix region of the AM after trauma. Figure 4 shows representative images of the AM control region with evidence of collagen fibril bundle organisation through the compact, fibroblast and spongy layers that appeared compact, irregular and basket‐like. In contrast, this arrangement changes in wounded AM within the stromal layers with evidence of collagen fibrils that are organised and aligned in parallel to the axis of the wound edge at 24 and 168 h of culture, similar to that observed with cell polarisation at the wound edge (Figure 1F–H). At the defect site, the direction of collagen fibre organisation showed a region of highly polarised fibres in all regions of the wound edge with a spread of ~0° (bottom panel, Figure 4). Bottom panel shows the formation of the collagen fibres, which appear dense, elongated and highly aligned with evidence of greater intensity of the SHG signal close to the wound edge. This collagen structure appears to be more coherent and presents a different profile to control AM where the fibre arrangement is disorganised and interwoven in a random fashion.

### Cell viability assessment of the fetal membrane defect at the trauma site

**Figure 5:**
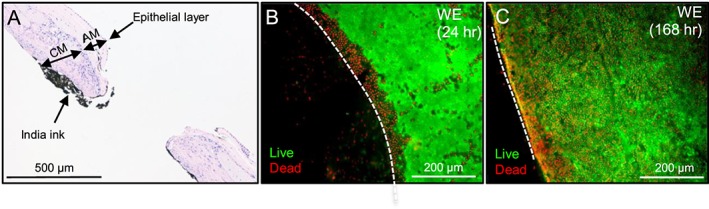
Histological examination and cell viability of the fetal membrane defect. H&E staining of the cross sections shows the different layers of the fetal membrane defect 24 h after trauma (A). India ink was used to locate the wound edge (WE), which shows a 0.8 mm lesion with no observed damage to the integrity of the amniotic membrane (AM) and chorionic membrane (CM) after 24 h. Cell viability analysis by live/dead staining shows viable cells (green) at 24 (B) and 168 h (C) away from the wound. However, there was a band of non‐viable cells (red) distinguishable within 100 μm of the WE primarily after 24 and 168 h. The dotted white lines show the border along the length of the WE

Histological cross sections of the FM show a wound edge region located with India ink staining (Figure 5A). We observed visible AM and CM regions in the FM tissues with the cell layers clearly defined with H&E staining. Confocal microscopy showed evidence of cell death along the wound edge of the FM defect after trauma compared to a region of viable cells away from the edge of the wound (Figure 5B and C).

## Discussion

The present study developed an artificial FM trauma model to examine the changes of Cx43 expression and collagen architecture in the AM *in vitro*. In this study, we chose the AM for evaluation of the FM defect because Cx43 was previously shown to be altered in the fibroblast and epithelial layers.^27^ Whilst several studies have described the use of synthetic materials or medical adhesives as promising agents to seal FM defects, tissue regeneration has been variable.^8^, ^9^, ^11^, ^12^, ^13^, ^14^, ^15^, ^16^ One difficulty is that there is no ideal model which allows examination of the synthetic material in a challenging wet and highly charged AF microenvironment.^29^ In addition, agents that knock down Cx43 have been shown to be effective in promoting healing properties and regeneration of skin wounds in rodents, implying that applications that can improve cell migration and repair the FM defect should be explored further.

We examined cell behaviour in wound edge FM and observed differences in cell shape in the AM compared to membranes taken away from the wound in patient matched controls. The differences in cell morphology were closely associated with enhanced expression of Cx43, which we found to be highly expressed by mesenchymal cells present in the fibroblast layer. This region also showed punctate Cx43 plaques with cells that had polarised 90° to the wound edge in identical fashion to the alignment of collagen observed. However, the efficiency of cell migration across the defect was poor as evidenced by the layer of cells on the edge and outer regions of the wound. The reason for the low efficacy of cell migration could be attributed to the overexpression of Cx43. Indeed, previous models of human chronic skin wounds have shown spatial and temporal expression of connexin proteins with specific amplification of Cx43 expression at the wound edge, and this protein has been shown to slow down healing.^25^, ^26^, ^30^ These studies also identified that the re‐epithelialization was characterised by an initial Cx43 downregulation at the wound edge with no expression detected within cells of the leading edge prior to migration, which is a similar observation to the present study which showed high levels of Cx43 in wound edge AM. However, the link between Cx43 expression and tissue healing has not been established in fetal membranes. It is also not known whether pre‐term membranes will behave in a similar way to TERM tissues. Our observations therefore need to be studied in large groups of patients and with much more detail of the variables (e.g. maternal age, gestational age, tissue location). However, the presence of very high levels of Cx43 protein within the epidermal and dermal wound edges of venous leg ulcers and diabetic skin wounds was shown to be a specific inhibitor of keratinocyte and fibroblast migration. These cells play a significant role in tissue contraction during wound closure. It is now apparent that downregulation of Cx43 in 3T3 fibroblasts with Cx43 antisense oligodeoxynucleotdide application or short hairpin RNA transduction results in significantly faster rates of migration in scratch wound assays.^31^, ^32^ Conversely, overexpression of Cx43 is associated with the opposite effect. Increasing Cx43 expression by pharmacological agents or transfection with Cx43‐GFP results in repressed production of lamellipodia and retarded migration. The overexpression of Cx43 in the AM after introduction of a wound in the FM has the potential to interfere with normal healing. Whilst Cx43 expression remains high in the proliferating cells away from the wound edge, it is likely that healing stalls in wounded cells and the mechanisms to induce healing should be explored further.

We used SHG imaging to study high‐resolution collagen organisation within the AM. Control AM showed minimal collagen alignment compared to the wound edge which appeared highly aligned within the stromal layers. Collagen is well known to contribute to the mechanical strength of the AM.^33^ However, the process of open wound healing by wound contraction mechanisms is complex. For example, thicker collagen fibres and denser regions of granulation tissue were noted at the wound edge in mature contracting skin wounds compared to younger wounds.^34^ In non‐healing chronic skin wounds, collagen bundles were found to lie tangential to the surface.^35^ Interestingly, embryonic wound healing involves the unique formation of a filamentous actomyosin ring at the wound margin, which enables wound closure by a process known as ‘purse‐string’ contraction that brings the wound edges together.^36^, ^37^, ^38^ It is possible that the cells in the present study align in a tangential fashion in order to join up via focal adhesions in an attempt to close the wound.^39^ However, this ‘purse‐string’ contraction should be followed by lamellipodial and filopodial protrusions towards the wound centre that facilitates the restoration of a continuous epithelial layer which is not seen in the present study.^40^, ^41^, ^42^, ^43^ The overexpression of Cx43 may interfere with the purse‐string contraction mechanism leading to an absence of wound closure, which warrants further investigation.

In summary, the present study showed that Cx43 overexpression in wounded AM drives structural changes in collagen that slows down efficacy of cell migration across the FM defect. The correlation between Cx43 and wound healing mechanisms, as previously shown in chronic skin wounds where Cx43 is overexpressed and healing is perturbed, is an approach that should be explored to repair the fetal membrane defect. This work has clear relevance to clinicians and their patients to prevent preterm birth after iatrogenic PPROM and could translate into guidelines for therapy protocols.
